# miRSel: Automated extraction of associations between microRNAs and genes from the biomedical literature

**DOI:** 10.1186/1471-2105-11-135

**Published:** 2010-03-16

**Authors:** Haroon Naeem, Robert Küffner, Gergely Csaba, Ralf Zimmer

**Affiliations:** 1 Institut für Informatik, Ludwig-Maximilians-Universität München, Amalienstr. 17 80333 München, Germany

## Abstract

**Background:**

MicroRNAs have been discovered as important regulators of gene expression. To identify the target genes of microRNAs, several databases and prediction algorithms have been developed. Only few experimentally confirmed microRNA targets are available in databases. Many of the microRNA targets stored in databases were derived from large-scale experiments that are considered not very reliable. We propose to use text mining of publication abstracts for extracting microRNA-gene associations including microRNA-target relations to complement current repositories.

**Results:**

The microRNA-gene association database miRSel combines text-mining results with existing databases and computational predictions. Text mining enables the reliable extraction of microRNA, gene and protein occurrences as well as their relationships from texts. Thereby, we increased the number of human, mouse and rat miRNA-gene associations by at least three-fold as compared to e.g. TarBase, a resource for miRNA-gene associations.

**Conclusions:**

Our database miRSel offers the currently largest collection of literature derived miRNA-gene associations. Comprehensive collections of miRNA-gene associations are important for the development of miRNA target prediction tools and the analysis of regulatory networks. miRSel is updated daily and can be queried using a web-based interface via microRNA identifiers, gene and protein names, PubMed queries as well as gene ontology (GO) terms. miRSel is freely available online at http://services.bio.ifi.lmu.de/mirsel.

## Background

MicroRNAs (miRNAs) are small (~22-nucleotide) non-coding RNAs (ncRNAs) that post-transcriptionally regulate the levels of a potentially large number of proteins by base-pairing to messenger RNAs (mRNAs) [1,2]. Perfect or near-perfect complementarity to the target RNA promotes cleavage and degradation of the RNA, while imperfect base pairing impairs translation of the target mRNA [3,4]. Functional studies implicate effects of miRNAs on a wide range of cellular and developmental processes such as cell cycle control, cell growth, apoptosis, embryo development, stress response, metabolism or morphogenesis and in various diseases including cancer [5,6].

Since the discovery of the first miRNA, lin-4 in *Caenorhabditis elegans *[7], thousands of miRNAs have been identified in vertebrates, flies, worms and plants and even in viruses [8]. Tens of thousands of gene targets have been predicted mostly by the use of automatic prediction algorithms such as [9-15]. So far, the targets of only a handful of these miRNAs have been experimentally validated [16-18]. Recently developed databases provide resources of miRNA nomenclature, sequence data, genomic localization and functional annotation in human, mouse, rat and other organisms [7,19,20] similar to the established gene-specific databases [21-24]. Several web-based tools integrate predicted miRNA targets e.g. miRNAMap 2.0 [25], miRGator [19], miRGen [20]. The databases miR2Disease [5], miRecords [16] and TarBase [18] collect target genes of the miRNAs in different organisms. Most miRNA-target associations contained in these databases are derived from the large-scale experiments where a detailed experimental validation of individual pairs has not been performed. For instance, TarBase and miRecords report 1031 and 776 miRNA-target pairs in human respectively. Out of these, 769 and 447 have been obtained from the supplementary material of just two publications [26,27] and [27,28]. miRecords additionally collects 158 rat miRNA-target gene pairs, 140 out of these pairs are extracted from a single publication [29]. A more detailed analysis has been performed by Ritchie et al [30]. They found that only 48 miRNA-target pairs of miRecords are sufficiently validated by experiments and, as a consequence, they conclude that benchmarks for the evaluation of miRNA target prediction algorithms cannot be constructed from the available databases.

As a remedy to this, information extraction applied to the scientific literature can be used to significantly enhance and complement the information stored in biological databases [31-36]. We therefore analyze PubMed abstracts to extract miRNA-gene associations. This includes generic miRNA-target relations but also five different kinds of specific miRNA-gene relations (physical target, co-expression, repression, induction, and cleavage) for our database miRSel. The manuscript gives a brief introduction into miRNA, gene and protein naming. It then describes the steps required for the implementation, population and evaluation of the miRSel database. We also provide a web-interface for querying and analyzing the database.

### miRNA, gene and protein naming conventions

The nomenclature of miRNAs and especially proteins as well as genes has evolved over time [7,32] and various naming conventions have been and are used in databases and in the scientific literature. For genes and proteins but also miRNAs typically several synonyms are in use. Unfortunately, synonyms often overlap with other synonyms (of other objects) or with names and abbreviations for diseases, species, experimental techniques, and even general English words [32,33]. For instance, for the gene ADCY10 (Entrez Gene identifier 55811) more than 10 additional synonyms are known not taking into account orthographical variations, such as usage of hyphens and slashes [32-36]. In comparison, miRNAs naming conventions have been described early and appear to be quite simple as miRNA names are based on sequential numerical identifiers (e.g., miR-1, miR-2 ... miR-101, etc.) and a prefixed species identifier (e.g. hsa-miR-100) [7,37,38].

The following conventions for miRNA naming are used:

(i) The predicted stem-loop portion of the primary transcript is named by a 3 or 4 letter species prefix and a numerical suffix (e.g. hsa-mir-100 in Homo sapiens). Whereas, the name of the excised ~22 nucleotide sequence (mature miRNA) contains the same mir, prefix and suffix as stem-loop but with capital miR (e.g. hsa-miR-100).

(ii) Orthologous miRNA sequences in different species are assigned the same names (e.g. mmu-miR-100 in Mus musculus, rno-miR-100 in Rattus norvegicus).

(iii)Mature miRNA sequences can be expressed from each arm of the hairpin precursor. They are distinguished by additional suffixes (e.g. hsa-miR-1224-5p (5'arm) and hsa-miR-1224-3p (3'arm)). Previously, they also have been named for instance miR-142-s (5'arm) and miR-142-as (3'arm). In some cases, the asterisk has been used to denote the less predominant form (e.g. hsa-miR-100*).

(iv) Distinct hairpin loci in a given organism that give rise to identical mature miRNA sequences are assigned names with additional numeric suffixes (e.g. hsa-mir-101-1 and hsa-mir-101-2 indicating two genomic loci of the miRNA hsa-miR-101).

(v) Related hairpin loci that give rise to related mature miRNA sequences with only one or two base changes are assigned letter suffixes of the form (e.g. hsa-mir-10a and hsa-mir-10b are similar sequences).

Of course, these conventions are not strictly followed in scientific publications. If complete names are used, e.g. hsa-miR-1224-5p, the author likely means the 5'arm predominant mature form of human miRNA-1224. On the other hand, an incomplete form e.g. miR-1224 could mean precursor or mature microRNAs, the 3' or the 5' variant or an unspecified variant of microRNA 1224 in some species depending on the context.

In addition, there are many naming problems: For some organisms fairly different naming conventions are used [7,37]. For instance, in plants, miRNA names are of the form MIR472 (in Arabidopsis thaliana) and only letter suffixes are used to represent distinct hairpin loci expressing related mature miRNA sequences [7]. Viral miRNAs names are based on the gene locus from which the miRNAs derive (e.g. ebv-mir-BARTT8 is a miRNA from BART locus of the Epstein-Barr virus) [7]. Capitalisation of names should not be relied on to confer information, such as mir and miR distinguishing between precursor and mature forms [37]. lin-4 and let-7 miRNAs are the apparent exceptions to the generic scheme [7].

## Implementation

The construction of a database of miRNA-gene co-occurrences via named entity recognition (NER) requires the compilation of miRNA, gene and protein name dictionaries as well as their association to database identifiers. The extraction performance depends on the completeness and uniqueness of the entries in the dictionaries. The dictionaries for human, mouse and rat are compiled from several databases: HUGO Gene Nomenclature Committee (HGNC) [21], Mouse Genome Database (MGD) [22], gene-centered information at NCBI (Entrez Gene) [23], Swiss-Prot Protein Database (Swiss-Prot) [24], miRGen [20] and miRBase [7]. The names, aliases, symbols, official names, synonyms, abbreviations, and database identifiers of proteins, genes and miRNAs from these databases have been merged into synonym dictionaries.

The next steps are extension and curation of the dictionaries. For proteins, we first complement the synonym lists with spelling variants, acronyms, abbreviations and long forms (e.g. *IL *= *Interleukin*). Secondly, inappropriate synonyms or expressions that would lead to ambiguous or wrong identifications are identified and removed. A detailed description of the curation and the involved processing steps is given in [32,33].

In case of miRNAs we found that many miRNA names described in the literature are not yet contained in databases. Therefore, we detect miRNA names using a regular expression. This regular expression has been constructed to match all database contained synonyms and generic occurrences of miRNA names as described in the section on miRNA naming conventions including species specific conventions (e.g. HUGO). The regular expression also covers frequent spelling variants mentioned in the texts (e.g. miR101b, miRNA-101b, microRNA-101b, microRNA101b, etc.) with and without species identifiers (e.g. hsa-miR-195 and miR-195). If detected miRNA synonyms are contained in public databases we map them to their database identifiers and, if possible, distinguish matches as stem loop sequence, mature sequence and gene family matches in miRSel.

The database derived synonyms are summarized in Table 1. Only comparatively small numbers of distinct miRNA loci can actually be found in any PubMed abstract. In human, mouse and rat only 360 different miRNA loci were detected in miRNA-target pairs using the regular expression. Even less, only 280 different miRNA loci would have been detected based on database derived synonyms alone.

**Table 1:** miRNA and gene/protein dictionaries

				Proteins/genes	
Species	Mature (miR/miR*)	miRNA Stem-loop	Synonyms	Entities	Synonyms
**Human**	1026	162	43070	30120	473403
**Mouse**	767	133	32448	42130	460921
**Rat**	392	63	15662	39545	285483

The identifiers and synonyms are extracted from different biological databases (such as miRBase, miRGen, HUGO, MGI, Entrez Gene, Swiss-Prot), including manually collected miRNA identifiers for human, mouse and rat from the literature. All dictionaries were processed to add frequently used synonym variants and to remove unspecific and inappropriate synonyms.

Proteins and genes from the dictionaries are detected in texts (Named Entity Recognition, NER) by string matching using the newly developed tool syngrep [39]. syngrep uses the Aho-Corasick algorithm [40] for fast matching, tolerates small synonym variations, and uses context resolution techniques to avoid and resolve ambiguities. As mentioned above, miRNAs are matched using a regular expression. The scan of the organism specific miRNA, gene and protein synonym lists (more than 115K objects and 1.2 M synonyms) against the entire PubMed (19 M records, 66 Gb XML) requires about 30 minutes on a PC with 4 CPU-Cores.

The identification of named entities allows to identify miRNA-gene pairs both co-occurring in an abstract or in a single sentence. If not mentioned otherwise, we will focus on pairs extracted from single sentences as they are more reliable for extracting miRNA-gene associations. Information on relations or interactions between miRNA and genes is of interest for generating and analyzing network models of regulatory pathways. These pairs are extracted and stored in miRSel together with the PubMed abstracts and sentences where they have been found.

We further compiled a list of 70 terms that are used to describe relations of interest between miRNA-gene pairs (see the section in additional file 1). These 70 terms are indicative of five different types of relations, namely physical target, repression, co-expression, induction and cleavage. miRSel contains miRNA-gene associations of these five types which have been identified as tri-occurrences of a miRNA, a relation-term, and a gene or protein in a single sentence of PubMed abstracts. miRSel users can also retrieve abstract instead of sentence co-occurrences if recall is more important than precision.

The miRSel user interface allows to query occurrences, pairs and associations of miRNAs and genes and to restrict the entries in the database using a number of filter criteria:

• The strictness filter enforces a strict string matching of occurrences against the dictionary entries (i.e. occurrences with special characters not in the dictionary or wrong case are removed) (default selected).

• The single-sentence filter reports only miRNA and gene pairs co-occurring in single sentences as opposed to pairs co-occurring in abstracts (default abstract).

• The relation-type filter restricts matches to a particular type of miRNA-gene association (default none is selected).

• The taxonomy filter aims to enforce organism specificity of the matches. Our organism specific taxonomy dictionary contains synonyms and MeSH vocabularies for all examined organisms as provided by the NCBI taxonomy database [41]. We define organism specific matches as tri-occurrences of a gene name, a miRNA name and an entry of the taxonomy dictionary (default none is selected).

• The gene synonym filter excludes protein or gene synonyms which are assigned to multiple genes or proteins (ambiguous synonyms) in databases (default none is selected).

• The database filter shows the text mining pairs only if they also contained in other databases or computational predictions of miRNA gene targets (default none is selected).

**Figure 1:**
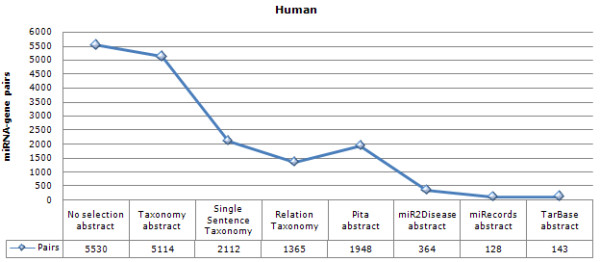
**The number of miRNA and gene/protein pair matches with synonym expansion, strictness and post filters in human**. **No selection **all miRNA-gene co-occurrences found in the publication titles and abstracts are displayed. Counts of miRNA-target pairs in the main text refer to this first column. The organism specificity can be increased by the **taxonomy **filter that requires confirmation of the selected organism. The text-mining results can also be restricted to miRNA gene pairs found within **single sentences**. The particlular type of association in miRNA-gene pairs can be restricted by the **relation **filter. Additional filters report pairs only if they are confirmed by target prediction algorithms (e.g. **Pita**) or manually curated databases (e.g. **miRecords, mir2Disease, TarBase**).

The total number of associations with regard to these filters is shown in Figure 1 for human.

## Results

### Web interface

**Figure 2:**
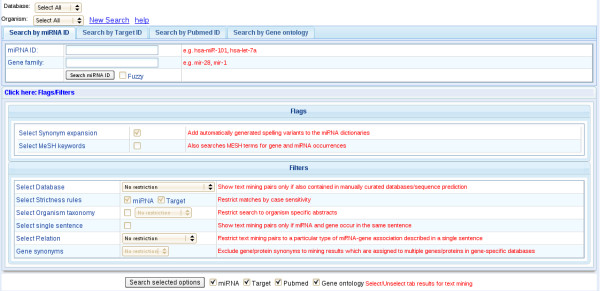
**A web based graphical user interface to the database**. miRSel can be queried via different options, including miRNA, target, gene ontology and PubMed keyword queries. If multiple options are selected, the results are AND-combined. Several filters are provided to control recall vs. precision of the mining results. For details see text.

miRSel provides a web interface to retrieve information on miRNA-gene pairs stored in the database (Figure 2). The interface allows to AND-combine different options to restrict query result sets.

(i) Genes can be selected based on gene names, gene symbols, protein names or database identifiers.

(ii) miRNAs can be selected based on miRNA identifiers and miRNA gene families.

(iii)A PubMed interface enables arbitrary PubMed keyword queries for searching miRSel, miRNA-gene pairs are reported only if found in PubMed abstracts matching the PubMed query.

(iv) The gene ontology (GO) option restricts the reported miRNA-gene pairs to genes associated with the selected GO-terms [42].

**Figure 3:**
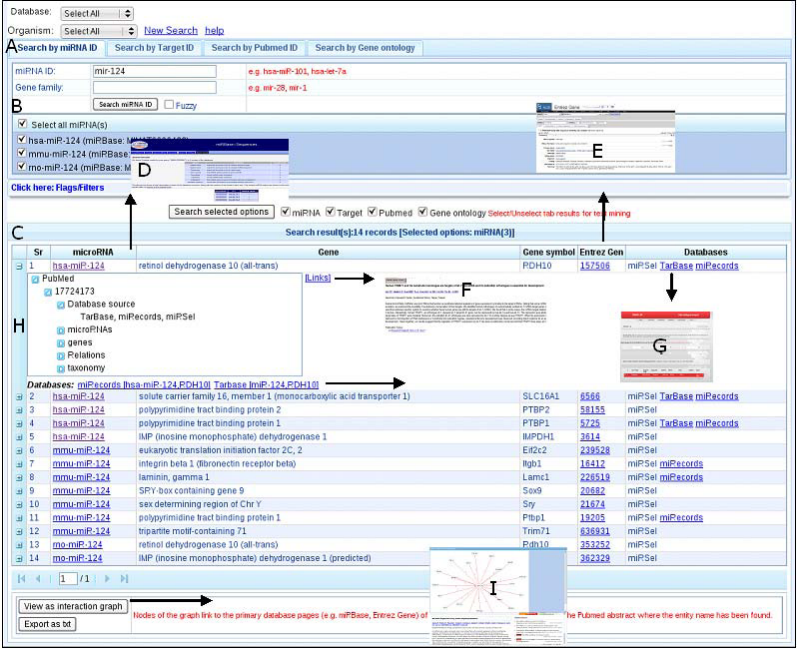
**A schematic workflow of miRSel search by miRNA ID**. After entering a complete or partial search key (e.g. a miRNA) (A) the user can select a subset of the matching miRNAs (B). Then, corresponding miRNA-target co-occurrences stored in the database are displayed in a tabular format (C). This table enables the navigation to miRNA or gene pages of primary databases (e.g. D = miRBase, E = Entrez Gene, PubMed abstracts that reference particular co-occurrences (F), or to the database sources for which the pair has been integrated (G). Also, details related to each miRNA-target pair e.g. all possible names for a given miRNA or protein in the literature and comparison results of other databases and sequence prediction can be displayed from the table (H). Finally, a miRNA target interaction graph (I) can be displayed that also enables the navigation to miRNA and gene pages (nodes) or PubMed abstracts (edges).

Additional filter options are described in the implementation section. Figure 2 shows the query mask and Figure 3 schematically depicts the query procedure. As a primary query result, an annotated table of miRNA-gene pairs is presented to the user. The table shows whether the pairs are contained in one of the manually curated databases (e.g. TarBase, miR2Disease) or if they have been predicted by miRNA-target prediction algorithms. Besides the table view, miRNA-gene pairs can be analyzed graphically using the Graphviz software [43] (Figure 3). Both representations provide links to the primary database pages (e.g. miRBase, Entrez Gene) of the found entities and to the PubMed abstracts where the entity names have been found.

### Evaluation

miRSel is based on finding occurrences of valid identifiers of genes, proteins and miRNAs in publication abstracts. Here we report on the performance of miRSel with respect to finding valid miRNA, gene and protein occurrences as well as valid miRNA-gene pairs and detailed miRNA-gene pair associations.

We estimate the reliability of the detection of miRNAs in texts in the following. The performance of gene and protein name detection has already been evaluated in the BioCreAtIvE competition [33].

For evaluation we selected PubMed abstracts that matched our regular expression for the detection of miRNAs or contained keywords such as 'microRNA', 'miRNA' 'mir', 'miR' and 'MIR'. Sentences containing a miRNA identifier or related keywords were additionally required to contain protein names from our synonym lists described in the implementation section. 50 PubMed abstracts were chosen randomly containing 89 sentences that met the above requirements. miRSel was compared against various manual analyses (see below) in terms of recall (i.e. fraction of True Positive (TP) and all True occurrences), precision (i.e. fraction of TP and all predictions) and f-measure.

The evaluation of miRNA identifier occurrences is shown in Table 2(a). Thanks to the regular expression based matching, the detection of miRNA identifiers in texts is very reliable.

**Table 2:** Evaluation of the detection of miRNAs and miRNA-gene associations.

Performance evaluation	abstracts	sentences	cases	Recall	precision	f-meas
**(a) miRNA occurrences**	50	89	79	0.96	1.00	0.98
**(b) miRNA-gene associations**	50	89	181	0.90	0.65	0.76
**(c) like b, after disambiguation**	50	89	181	0.88	0.78	0.83
**(d) like b, with keywords**	20	29	103	0.89	0.70	0.78
**(e) like b, association types**	20	29	103	0.87	0.62	0.73

For the detection of miRNA-gene associations we manually evaluated if a gene and a miRNA have been correctly detected my miRSel and if an association between the two is implied. As shown in Table 2(b), many of the pairs in miRSel represent valid associations. The detection of miRNA-gene associations has been further refined by automatically resolving ambiguities to gene identifiers by using additional tissue and cell-line dictionaries (Table 2(c)).

Besides the detection of generic miRNA-gene associations, miRSel automatically annotates five different types of associations between miRNAs and genes (physical target, co-expression, repression, induction, and cleavage; see implementation section for details). Out of the 1973 single-sentence human miRNA-gene pairs in miRSel 1301 (65%) were classified into one of the five types.

From the test set described above, a subset of the sentences that contain association keywords have also been evaluated manually. If association keywords are present in sentences with miRNA-gene pairs, the precision of association detection increases slightly (Table 2, compare b and d). If a true miRNA-gene association is detected, association keywords describe the type of association correctly in 89% of the cases (Table 2, compare d and e).

### miRSel Query Examples: p53 protein and hsa-miR-21

The TP53 gene (Entrez Gene: 7157) encodes protein p53, which is one of the most important tumor suppressor proteins. TarBase and miRecords do not report any miRNA targeting this gene. We extracted 60 different human miRNAs that co-occur with this target gene from 79 PubMed abstracts, and some of them (e.g. hsa-let-7a, hsa-miR-30b, hsa-miR-183) are consistent with microarray-based results discussed by Shalgi et al. [44].

hsa-miR-21 is the most frequent miRNA in miR2Disease, with 59 documented associations of this miRNA with diseases. miRSel contains 181 different genes co-occurring with this miRNA extracted from 123 PubMed abstracts. 96 pairs are retrieved if miRSel results are restricted to the more reliable single-sentence pairs.

## Discussion and Conclusion

miRNA related research depends on knowledge of miRNA target genes. The available databases on literature derived miRNA target pairs demonstrate that manual curation is difficult. TarBase, for instance, contains only 1135 (in human 1031, mouse 101 and rat 2) such pairs (TarBase version 5). Moreover, only a fraction of the content in current databases has been derived by manual curation of experimentally validated targets. Instead, the major part of the content stems from the supplemental material of few research articles describing large scale experiments. Ritchie et al [30] propose to exclude such studies for lack of a sufficient experimental validation. Only 262 out of 1135 miRNA-target pairs remain after excluding just two such studies from TarBase. In contrast to manual curation we proposed a simple, automated approach for biological name identification (named entity recognition, NER) that collects many potential targets for miRNAs not contained in current databases. Text mining of miRNA, gene or protein names results in good recall and precision for miRNA-gene associations detected in single sentences. We thereby extracted many pairs from human (2112 pairs), mouse (895 pairs) and rat (231 pairs) abstracts as well as 452 pairs from abstracts discussing other organisms. This represents an about 10-fold increase with respect to TarBase if miRNA target pairs derived from large scale experiments are excluded (a three fold increase as compared to the whole TarBase). miRSel also characterizes many miRNA target pairs with one of the five different association types. Here, 1365 in human (65% of single sentence pairs), 646 in mouse (72% of single sentence pairs), and 196 (83% of single sentence pairs) in rat have been thus annotated in miRSel. Such an annotation is also available from public databases, but only for very few pairs, e.g. 170 pairs in TarBase. miRSel can also provide 5314 pairs that co-occur in abstracts for human, 2178 for mouse and 505 for rat, which are expected to be less reliable compared to pairs derived from single sentences.

To keep the miRSel database up-to-date, newly available PubMed abstracts are included daily. A full refresh of the synonym list generation and, subsequently, the scan of the entire PubMed is performed monthly to ensure the validity of all identifiers.

Finally, we provide a web interface for querying miRSel via miRNA names, gene or protein names and via restricting the results using gene ontology terms or PubMed queries. We provide additional filter options, for instance to ensure the taxonomy context of matches. For future development we will extend miRSel to full free text and apply miRSel to additional species.

## Availability and requirements

**Project Name**: miRSel

**Project home page**: http://services.bio.ifi.lmu.de/mirsel

**Operating systems**: Windows, Linux

**Programming language**: Java (JDK 1.6.0, ZKoss framework), Tomcat v6.0

**License**: free

**Any restriction to use by non-academics**: None

## Authors' contributions

HN, RK, and RZ planned the project. HN acquired the data and performed validations. HN and GC implemented the database and web server. GC implemented the name entity system and HN processed and integrated the raw data. RK and HN analyzed and finalized the design and performance. HN, RK and RZ prepared the manuscript. RZ and RK supervised the project. All authors tested the database and interface. All authors approved the final manuscript.

## Supplementary Material

Additional file 1 **Keywords describing miRNA-target relationship types**. miRSel aims to categorize detected miRNA-target associations into five different relationship types. relation_keywords.txt maps relationship types to keywords. If miRSel detects such keywords together with a miRNA-target association the corresponding relationship type is assigned. Click here for file
